# Call Off the Dog(ma): M1/M2 Polarization Is Concurrent following Traumatic Brain Injury

**DOI:** 10.1371/journal.pone.0148001

**Published:** 2016-01-25

**Authors:** Josh M. Morganti, Lara-Kirstie Riparip, Susanna Rosi

**Affiliations:** 1 Brain and Spinal Injury Center, University of California San Francisco, San Francisco, CA, United States of America; 2 Departments of Physical Therapy and Rehabilitation Science, University of California San Francisco, San Francisco, CA, United States of America; 3 Neurological Surgery, University of California San Francisco, San Francisco, CA, United States of America; University of South Florida, UNITED STATES

## Abstract

Following the primary mechanical impact, traumatic brain injury (TBI) induces the simultaneous production of a variety of pro- and anti-inflammatory molecular mediators. Given the variety of cell types and their requisite expression of cognate receptors this creates a highly complex inflammatory milieu. Increasingly in neurotrauma research there has been an effort to define injury-induced inflammatory responses within the context of *in vitro* defined macrophage polarization phenotypes, known as “M1” and “M2”. Herein, we expand upon our previous work in a rodent model of TBI to show that the categorization of inflammatory response cannot be so easily delineated using this nomenclature. Specifically, we show that TBI elicited a wide spectrum of concurrent expression responses within both pro- and anti-inflammatory arms. Moreover, we show that the cells principally responsible for the production of these inflammatory mediators, microglia/macrophages, simultaneously express both “M1” and “M2” phenotypic markers. Overall, these data align with recent reports suggesting that microglia/macrophages cannot adequately switch to a polarized “M1-only” or “M2-only” phenotype, but display a mixed phenotype due to the complex signaling events surrounding them.

## Introduction

Consistently, neuroinflammation is an axiomatic physiological response following traumatic brain injury (TBI). This response is propagated by a variety of cells types in the injured brain via upregulation and release of soluble cellular constituents to the surrounding parenchyma [1, 2]. Principally, these constituents are produced by CNS-resident microglia as well as astrocytes, which perpetuate the activation of the innate immune response in the injured brain [3]. By nature this networked response is inherently complex given the multitude of factors and cognate receptors involved, however much attention and research has been devoted to defining this complex inflammatory response within the dichotomous and linear constraints of ‘M1’ versus ‘M2’ innate polarization phenotypes. Innate immune polarization was originally described using *in vitro* examinations of the differential effects of singular stimuli (e.g. LPS, IL-4, IFN) on macrophage gene expression [4]. The “M1/M2” nomenclature was later derived [5], and then expanded into several subgroups [6–9] to accommodate for an ever-increasing spectrum of stimuli and subsequent gene expression responses of *in vitro* macrophages. Altogether, this was an effort to group tissue macrophage response akin to the synchronized responses of polarized lymphocytes [5]. Ultimately, gene profiling techniques have recently elucidated that the stabile polarization states formed by lymphocytes [10] do not map sufficiently to macrophages, which are distinctly plastic by comparison [11–14]. More recently, a review by Martinez and Gordon [15] recapitulated these findings suggesting, *in vivo*, that the inflammatory milieu present in disease or injury requires these cells to react to a variety of stimuli simultaneously, shaping their responses in complex and perhaps mixed phenotypes.

In light of these recent findings, we profiled the inflammatory response of the brain in the context of simultaneous or mixed macrophage phenotypes following TBI. Herein, we used our rodent model of moderate TBI [16] to define the temporal inflammatory profiles of the injured brain through multiple time points following injury. To examine inflammatory response we examined over ninety targets spanning the M1/M2 macrophage inflammatory spectrum, curated from highly cited manuscripts focused on macrophage polarization [6, 8, 11, 12, 15, 17–21]. In the current study we show that TBI initiates a broad-spectrum inflammatory response, simultaneously up-regulating the expression of both “M1” and “M2” associated genes in the TBI-injured brain. Importantly, the concurrent expression of both activation states spanned multiple time points following injury, creating a common node of differential gene expression. Furthermore, we show that microglia/macrophages in the injured parenchyma mirror these responses antigenically via concurrent expression of M1/M2 markers on the same cell across multiple time points. Taken together, these data further draw into question the viability of a dichotomous system to define parenchymal macrophage response in TBI given the complex signaling milieu surrounding these cells and their Janus-faced plasticity.

## Materials

### Animals

All experiments were conducted in accordance with the National Institutes of Health Guide for the Care and Use of Laboratory Animals and were approved by the Institutional Animal Care and Use Committee of the University of California (San Francisco, CA). Adult 3-month-old *C57BL6/J* (wild type; WT) male mice were used for all experiments, purchased from Jackson Laboratories (Bar Harbor, ME). Mice were group housed in environmentally controlled conditions with reverse light cycle (12:12 h light:dark cycle at 21 ± 1°C) and provided food and water *ad libitum*.

### Surgical Procedure

Animals were anesthetized and maintained with 2.5% isofluorane with a non-rebreathing nose cone and passive exhaust system connected to a stereotaxic frame (David Kopf). Once animals were secured with non-traumatic ear bars, eye ointment was applied and their heads were cleared of any hair around the scalp. Following betadine application, a midline incision was made through the scalp. TBI was reproduced in the parietal lobe using the controlled cortical impact (CCI) model [22]. Mice received a craniectomy approximately 3.5mm in diameter using an electric microdrill with the center point determined by a digitally calibrated manipulator arm (Leica) to the coordinates anteroposterior, -2.0mm; mediolateral, +2.0mm, with respect to bregma. Explicit attention was paid to prevent damage to the dura during craniectomy; any animal in which the dura was disrupted, as assessed by excessive bleeding, was omitted from the study and replaced by another littermate. Following craniectomy, contusion was achieved using a 3.0mm convex tip attached to an electromagnetic impactor (Leica) mounted to the digitally calibrated manipulator arm. In order to impact flush with the natural curvature of the head/tissue, the manipulator arm was rotated 20° on the vertical axis. The parameters for impact were for a contusion depth of 0.95mm (from dura), velocity was constant at 4.0m/s and the impact was sustained for 300ms. Importantly, these injury parameters penetrated all layers of the cortex stopping short of disrupting the dorsal hippocampal structure (AP -2.0mm; ML +2.0mm; DV -.95mm). Following CCI injury, the scalp was sutured and each animal received 0.5mL of physiologic saline (i.p.) before being placed in a water-heated incubation chamber (37°C) until they fully recovered as exhibited by resumption of movement and grooming. Sham animals were treated to the above parameters except that the CCI injury was omitted. Animals were euthanized exactly at 1 day (n = 10), 2 days (n = 10), and 7 days (n = 10) after surgery.

### qRT-PCR

For qRT-PCR gene expression endpoints all mice were euthanized via rapid cervical dislocation; brain tissues were quickly removed from the skull for dissection. Ipsilateral cortical tissue comprising the perilesional site and analogous area in sham mice were dissected and rapidly frozen in liquid nitrogen before long-term storage at -80°C. For RNA isolation, ipsilateral cortical tissues were homogenized in Trizol lysis buffer (Invitrogen) using disposable plastic pestle in 1.5mL microcentrifuge tubes. RNA was isolated using DirectZol columns (Zymo) with on-column DNAse treatment according to manufacturer’s instructions. RNA concentration and quality were determined using a NanoDrop Lite (Thermo Scientific). 300ng of RNA was reverse transcribed using High-Capacity cDNA Reverse Transcription Kit (Applied Biosystems). For inflammatory profiling arrays (Qiagen, #330131) equal volumes of cDNA for each sample were pooled and run on a single plate per condition (e.g. sham, 1d, 2d, and 7d); cycling conditions were followed as suggested by manufacturer. Selected targets from the profiling arrays were validated using individual samples: *ARG1* (GAACACGGCAGTGGCTTTAAC/TGCTTAGCTCTGTCTGCTTTGC), *B-Actin* (ACCCACACTGTGCCCATCTACG/GCCACGCTCGGTCAGGATCTTC), *CCL2* (GCTGACCCCAAGAAGGAATG/GTGCTTGAGGTGGTTGTGGA), *CCL3* (ACTGCCTGCTGCTTCTCCTACA/ATGACACCTGGCTGGGAGCAAA), *CCL4* (ACCCTCCCACTTCCTGCTGTTT/CTGTCTGCCTCTTTTGGTCAGG), *CCL5* (CCTGCTGCTTTGCCTACCTCTC/ACACACTTGGCGGTTCCTTCGA), *CCL7* (CAGAAGGATCACCAGTAGTCGG/ATAGCCTCCTCGACCCACTTCT), *CCL12* (CAGTCCTCAGGTATTGGCTGGA/TCCTTGGGGTCAGCACAGAT), *CD36* (GGACATTGAGATTCTTTTCCTCTG/GCAAAGGCATTGGCTGGAAGAAC), *CX3CL1* (GGCTAAGCCTCAGAGCATTG/CTGTAGTGGAGGGGGACTCA), *CXCL2* (CATCCAGAGCTTGAGTGTGACG/GGCTTCAGGGTCAAGGCAAACT), *CXCL3* (TGAGACCATCCAGAGCTTGACG/CCTTGGGGGTTGAGGCAAACTT), *CXCL10* (CCTCATCCTGCTGGGTCTG/CTCAACACGTGGGCAGGA), *CXCL12* (GGAGGATAGATGTGCTCTGGAAC/AGTGAGGATGGAGACCGTGGTG), *DUSP1* (CAACCACAAGGCAGACATCAGC/ GTAAGCAAGGCAGATGGTGGCT), *GM-CSF* (ACCACCTATGCGGATTTCAT/TCATTACGCAGGCACAAAAG), *IL1B* (TGTAATGAAAGACGGCACACC /TCTTCTTTGGGTATTGCTTGG), *IL4R* (ACCAGATGGAACTGTGGGCTGA/AGCAGCCATTCGTCGGACACAT), *PTX3* (CGAAATAGACAATGGACTTCATCC/CATCTGCGAGTTCTCCAGCATG), *TGB1* (TGATACGCCTGAGTGGCTGTCT/CACAAGAGCAGTGAGCGCTGAA), *TGM2* (GAAGGAACACGGCTGTCAGCAA/ GATGAGCAGGTTGCTGTTCTGG), *TLR1* (GGTAGCAAGAGAAGTGGTGGAG/ CGATGGTGACAGTCAGCAGAAC), *TLR2* (ACAGCAAGGTCTTCCTGGTTCC/GCTCCCTTACAGGCTGAGTTCT), *TLR8* (AAGTGCTGGACCTGAGCCACAA/CCTCTGTGAGGGTGTAAATGCC), *TNFA* (TGCCTATGTCTCAGCCTCTTC/GAGGCCATTTGGGAACTTCT). Amplifications of these gene transcripts were carried out in duplicate using SYBR Green Master mix (Applied Biosystems). All primer pairs were independently validated using a standard curve of serially diluted mouse cDNA before use in any endpoint. In each PCR analysis, template and RT controls were included to account for contamination. Cycling conditions were run as previously described [23]. Gene expression was normalized to beta-actin for all samples; fold change per condition was calculated using the 2^-ΔΔCt^ method, with sham group as the reference.

### Immunofluorescence Staining and Imaging

Mice were lethally overdosed using a mixture of ketamine (150 mg/kg)/xylazine (15 mg/kg). Each animal was transcardially perfused with phosphate-buffered saline (PBS), followed by 4% paraformaldehyde in PBS. Brain tissues were carefully removed and post-fixed in 4% paraformaldehyde for 16h at 4°C., after which they were transferred into 30% sucrose in PBS for another 16h at 4°C. Coronal sections were made at 40μm using a Microm cryostat (Richard-Allan Scientific) spanning the perilesional cortex. Standard staining procedures [24] were conducted on free-floating sections (n = 4-6/animal) using the following primary antibodies; CD68 (1:100, BioRad, MCA1957, lot#0812), MARCO (1:100, BioRad, MCA1849T, lot#0315), CD36 (1:100, BioRad, MCA2748GA, lot#0115), F4/80 (1:100, MCA497R, lot#1112), CD206 (1:100, BioRad, MCA2235, lot#0812), Iba1 (1:500, Wako, 019–19741, lot#CTR6026), Ym1 (1:100, StemCell Technologies, 14014, lot#13C48567). Detection of primary antibodies was achieved using appropriate conjugated secondary antibodies; anti-rabbit AF647 (1:200, Life Technologies, A21244, lot#1654324), anti-rat AF488 (1:200, Life Technologies, A11006, lot#1605895), and anti-rat 570 Fab (1:50, Jackson ImmunoResearch, 112-297-003, lot#85985). Following standard procedure, [24] serial incubations were conducted with each primary overnight at 4°C. Briefly, labeling was conducted using a sequential process such that a combination of a rabbit and rat antibody were incubated simultaneously overnight, following a series of washes and blocking as previously described [24], the primary-labeled antigens were revealed with anti-rabbit AF647 dye and the anti-rat 570 Fab secondaries for 2 hours at room temperature. Following a series of washes as described above, the second rat-host antibody was applied for overnight incubation at 4°C, which was revealed in the same manner above but with the anti-rat AF488 secondary. Nuclei were counterstained using DAPI (Invitrogen, D1306). Stained sections were mounted onto Superfrost Plus slides (Fisher, 12-550-15) and coverslipped with fluorescent mounting medium (Vector, H1000). Explicit care was taken to confirm the specificity of all antibody combinations by using ‘no-primary’ controls for each staining condition. Images were acquired using a Zeiss Imager.Z1 Apotome microscope controlled by Zen software (Zeiss 2014) for single plane images and the Zeiss LSM780 for confocal images. Only linear adjustments were made during image acquisition. All images were acquired immediately adjacent to the CCI-induced cavitation in the gray matter.

### Data Analysis

Target genes of interest were determined by examining responses representing ± 1.5 fold change or greater, relative to sham. Comparative analysis was generated in Ingenuity IPA software (Qiagen) using simple-set theory to render common overlapping nodes among the three time points. Proportional Venn diagram was generated using euler*APE* [25]. Expression of single gene targets was analyzed using one-way ANOVA with Dunnett’s posthoc test for multiple comparisons in GraphPad Prism software (v6.0d; La Jolla, CA). Significance was set to p<0.05.

## Results

### Morphological Characterization of microglia/Macrophages Is Not Sufficient to Determine Inflammatory Profile

**Fig 1:**
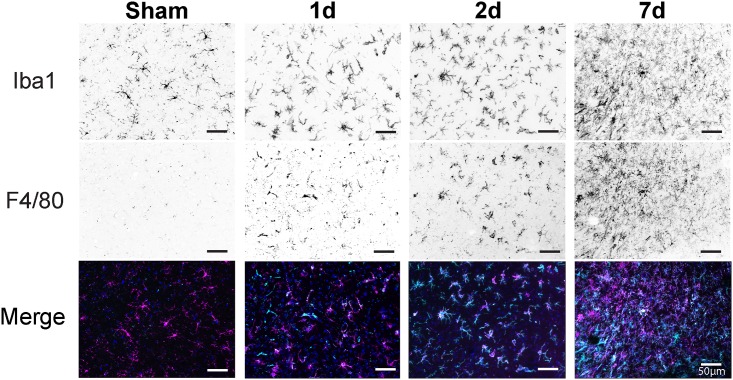
Morphology of microglia/macrophages after TBI across time. Representative images of perilesion from sham and injured animals were stained using pan markers for microglia (Iba1) and microglia/macrophages (F4/80) and visualized with fluorescently conjugated antibodies. Sham animals displayed the morphology of “resting” microglia with compact soma and long, skinny processes. There were a few dual labeled Iba1^+^ microglia with a punctate labeling of F4/80 in the sham animals. Comparatively, there was a drastic change in morphology as a response to injury and across time for both markers. Injury induced a visual increase in the number of F4/80^+^ cells as a progression of time. Representative pseudocolored images were taken adjacent to the contusion site and converted to black and white, with black representing positive staining for each respective marker as labeled. Merged fluorescent images show the degree of coalescence for both markers. Iba1 = magenta, F4/80 = aquamarine.

**Fig 2:**
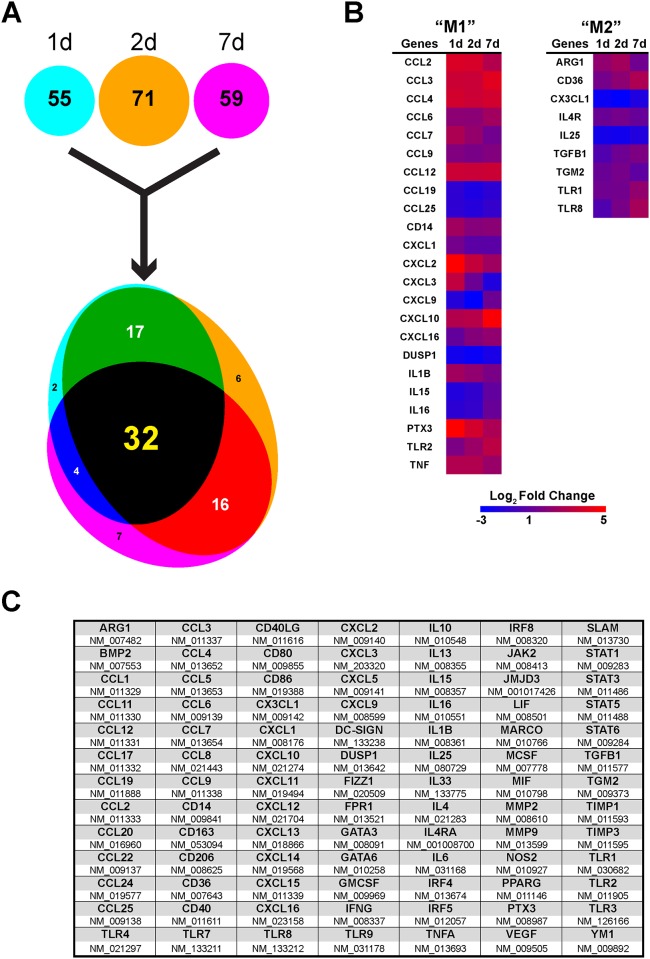
Gene profiling of inflammatory response after TBI across time. qRT-PCR gene profiling of pooled ipsilateral perilesion cortices after injury. (**A)** Using a custom curated qRT-PCR mini array of 91 genes identified 55, 71, and 59 genes of interest with a fold change greater than 1.5 over sham values for 1d, 2d, and 7d, respectively (n = 8/group). These genes were then grouped using simple-set theory, displayed graphically as a proportional Venn diagram. Therein, there were multiple overlapping nodes between each time point. The greatest degree of commonly expressed genes was shared with the 2d time point between both 1d and 7d. Comparatively, there were relatively few commonly expressed genes between 1 and 7 days. Importantly, across all time points there were 32 commonly expressed genes as response to TBI. (**B)** Representative heatmap of Log_2_ normalized fold changes for the 32 common-node genes. (**C)** Gene table for all genes in the curated profiling array. Bold items indicate gene name, with their respective RefSeq identifier listed immediately below. Red = upregulated, Blue = downregulated gene expression.

In the young, healthy CNS microglia display quite a dynamic capability [26] in an effort to survey their local microenvironment [27], however upon injury these highly ramified cells contract their processes in a visibly altered morphological arrangement. We labeled injury-induced alterations in microglia/macrophage morphology using a combination panel of pan-markers for microglia and macrophages, Iba1 and F4/80, respectively. TBI induced substantial morphological changes in the innate effectors of the parenchyma at each time point following injury (Fig 1). Moreover, as a progression of time after injury, there was a visual increase in coalescence of co-labeling in terms of these two pan markers for microglia/macrophages, which we have recently shown via flow cytometry [16]. We profiled ipsilateral perilesional cortical tissue (ipsi-Ctx) over three time points following injury; one day (1d), two days (2d), and 7 days (7d). Whole tissue was examined to define the inflammatory milieu in this perilesional environment. We set an arbitrary fold change cutoff such that only gene targets whose expression was greater than ±1.5 fold change were considered. Cumulatively, this filtered gene responses to yield 55, 71, and 59 differentially regulated genes for 1d, 2d, and 7d groups, respectively (Fig 2A). These genes were compared to determine common expression patterns, which yielded a common-node of 32 genes similarly expressed among the three time points; black zone of proportional Venn diagram (Fig 2A). Proportionally, there were more differentially expressed genes in common across all time points than any other inter-node. Comparatively, there was a spillover of gene products as a progression of time such that 48h, being the intermediate, had the most genes in common with 24h and 7d. While there were relatively few differentially expressed genes in common between 24h and 7 d. Importantly, for the common-node both pro- and anti-inflammatory mediators were differentially expressed across all time points (Fig 2B).

### Simultaneous M1/M2 Profiles Induced by TBI

**Fig 3:**
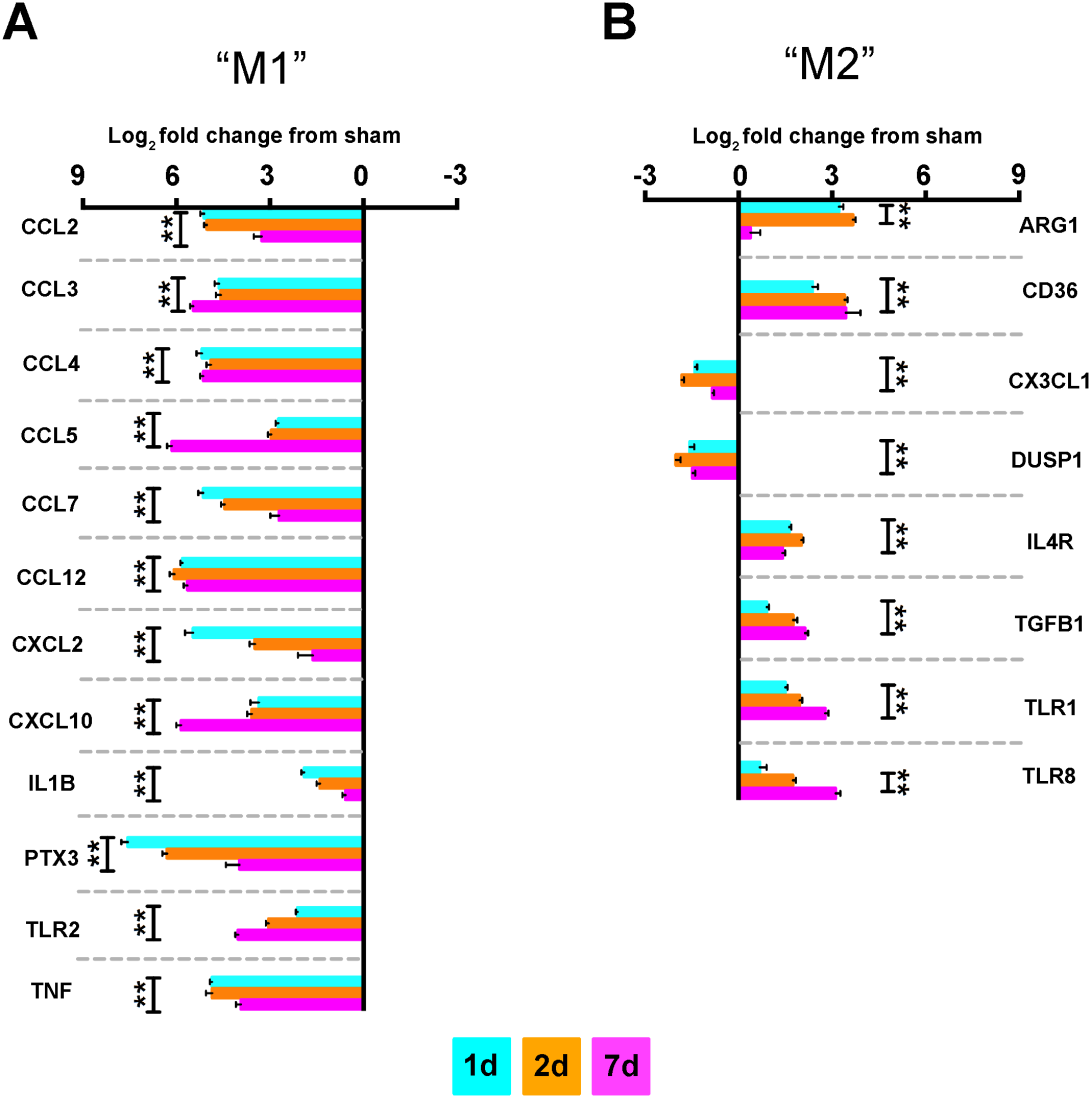
Concomitant expression of pro- and anti-inflammatory genes after TBI. Selected single gene expression analyses from TBI common-node. (**A/B)** Select genes comprising both pro- and anti-inflammatory mediators were validated using n = 8/time point, including sham. Log_2_ normalized fold change of these genes shows markedly similar expression profiles as produced by the pooled samples examined in the array. Of the genes displayed, all time points examined had significant differential expression, relative to sham, for each analyte except for *Arg1* at 7d and *TLR8* at 1d. Data are displayed as Log_2_ normalized fold change relative to sham + SEM. Sham is equivalent to 0. Data were analyzed using one-way ANOVA with Dunnett’s posthoc test for multiple comparisons wherein each mean is compared to the mean of the sham group. **p<0.01. Dumbbell-style brackets indicate same level of significance for each group relative to sham. 1d = aquamarine, 2d = orange, 7d = magenta.

**Fig 4:**
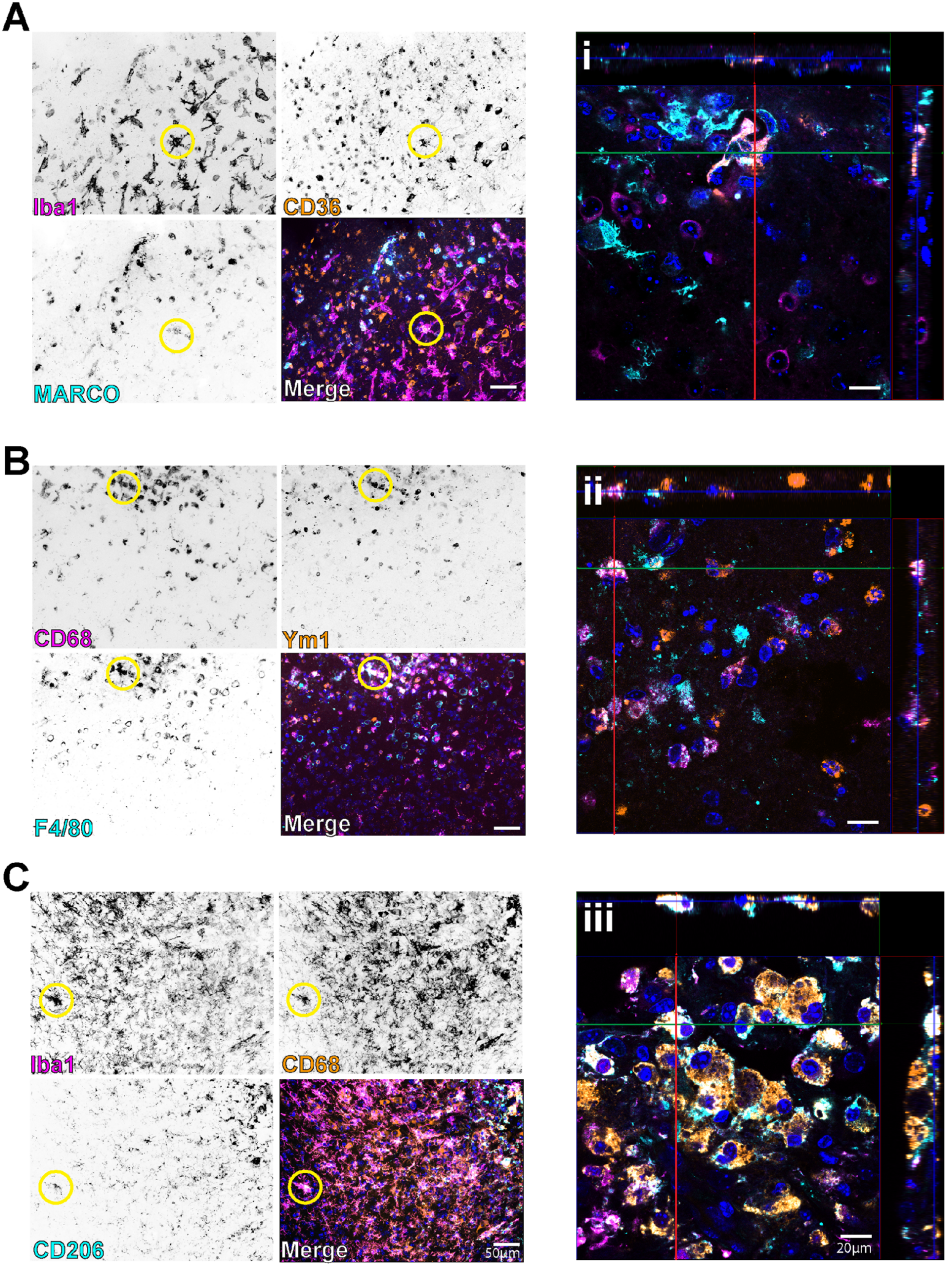
Simultaneous expression of M1/M2 markers in microglia/macrophages after TBI. Representative images of the three time points following injury, sequentially labeled for M1 and M2 markers. (**A/i)** 1d. (**B/ii)** 2d. (**C/iii)** 7d. The lower left hand corner of each representative image indicates the respectively labeled marker and pseudocolor for that image when viewed in the “merge” panel, with “merge” indicating that all three pseudocolored images are coalesced. (**i-iii)** High magnification confocal images of representative colocalized staining for each adjacent staining; pseudocolors are kept consistent between single plane images and confocal z-stacks, for clarity. Yellow circles indicate representative cells of interest displaying simultaneous labeling of all three markers for each panel. Iba1 = pan marker for microglial/macrophages, CD36 = M2 marker, MARCO = M1 marker, CD68 = M1/lysomal macrophage, Ym1 = M2 marker, F4/80 = pan marker for macrophages, CD206 = M2 marker. All representative images were taken adjacent to the contusion site. Pseudocolored images were converted to black and white for single markers, with black representing positive staining for each respective marker as labeled.

We next validated several selected targets from the common-node for each composite time point analyzed in the mini arrays. Very closely mirroring the pooled responses in the mini arrays, TBI initiated a heterogeneous inflammatory response via differential expression of both pro- and anti-inflammatory genes. At each time point, TBI induced significant expression changes of each analyte for both pro- (Fig 3A) and anti-inflammatory (Fig 3B) gene markers. Although each gene was significantly affected by TBI, there were some expression differences across time, comparatively, as we have previously shown [16]. Given the overlapping expression of a variety of inflammatory markers, we next examined if the mixed inflammatory phenotype was present on microglia/macrophages. Using the same time course, we co-labeled microglia/macrophages (Fig 1) with commonly used antigenic markers of “M1” or “M2” activation states. Herein, we show that that microglia/macrophages immediately adjacent to the CCI-induced cavitation display a mixed phenotype by co-labeling with both polarization markers across the three time points (Fig 4). Moreover, these techniques revealed a very heterogeneous parenchymal landscape such that the population of labeled cells visually created instances of dually labeled “M1/M2” cells along with a “M1” cell adjacent to “M2” cells.

## Discussion

Using a two-pronged approach, we highlighted the multifaceted inflammatory milieu of the parenchyma in the TBI-injured brain. With these methods we demonstrated that the binary categorization of “M1/M2” polarization phenotypes cannot be delineated in this injury model as both the microenvironment and within the same cell there is concurrent differential expression of both “M1” and “M2” phenotypes. Despite the commonly interpreted “activated” appearance of microglia/macrophages following injury; increased Iba1 and F4/80 staining, each time point examined showed marked heterogeneity in terms of gene expression as well as antigenic markers commonly associated with the *in vitro* established M1/M2 polarization paradigm. Common to each time point examined was the simultaneous expression of multiple chemokine signaling mechanisms involved in the induction, recruitment, and resolution of neuroinflammation, further highlighting the central and contributing role of these molecules in TBI-induced inflammation. Similarly, recent work using a reporter mouse model for Arginase1 expression paired with focal TBI showed that brain infiltrating macrophages, identified as Arg1+ or Arg1-, revealed concomitant gene expression of pro- and anti-inflammatory chemokine signatures [28], similar to overlapping inflammatory signatures of infiltrated macrophages in the injured spinal cord [29]. Moreover, in a diffuse injury model similar concurrent antigenic profiles were found on isolated microglia [30].

There is a great degree of concordance regarding the role of inflammatory response initiated by TBI between various animal models and human studies [31–36]. However, despite the highly complex inflammatory responses revealed by microarray [34, 37, 38] and bioinformatic approaches [16, 39], there is an increasing trend to adopt a binary approach to categorize these reactions based upon decade-old understanding of *in vitro*-derived stimulus responses of isolated macrophages [5]. However, if tissue resident microglia/macrophages are incapable of remaining in a tonically polarized phenotype [15] due to a lack of defined molecular mechanisms [11, 14], it becomes a problematic endeavor trying to maintain *in vitro* defined responses in animal models of disease [40]. Furthermore, these inconsistencies were recapitulated in two recent reviews by Martinez and Gordon [15] and Murray et al. [41].

Herein, we have purposefully expanded our previous observations that TBI induces inflammatory responses that do not neatly fall within the linear constraints of the M1/M2 paradigm [16]. Moreover, our current work is not the first to expand these findings as other models of neurotrauma have reported similar discord with this dogma [28, 30, 42–44]. Despite a variety of differentiating factors including time course, species, and injury location (e.g., brain or spinal cord) these studies [28, 30, 42–44] have shown that neurotrauma induces an inflammatory phenotype with simultaneous profiles. Admittedly, our work herein is by no means comprehensive in terms of examining every mediator previously reported to alter or represent M1/M2 bias. However, the targets examined comprise an ever-expanding repertoire of molecular mechanisms that are commonly reported in various animal models of neuroinflammation. Our data show that there is not a consistent bias towards one polarization phenotype versus another in this model. In the context of neurotrauma, these concomitant gene expression and antigenic profiles call into question the validity of examining a single antigenic marker of microglia/macrophage morphology (e.g., Iba1 and F4/80) to derive the inflammatory status of these cell populations. As such, our data show that although the microglia/macrophage population have acquired an ‘activated’ appearance, they are responding to both pro- and anti-inflammatory milieu simultaneously. Therefore, these markers of morphology must be used in the context of discerning, in a snapshot sense, the degree of perturbation of these cells and not as an index of inflammatory bias.

In conclusion, we have shown that the binary inflammatory phenotype of M1/M2 response is not clearly delineated in the context of neurotrauma. Similar to recent work in LPS-stimulated and ALS models of neuroinflammation [13] and spinal cord injury [29], we were unable to demonstrate an unambiguous inflammatory profile in both injured tissue and representative labeling of microglia/macrophages. Our findings support recent work acknowledging a disconnect between *in vitro* modeling of macrophage phenotypes and *in vivo* tissue response in disease [15, 40]. Importantly, our findings are not meant to discredit or draw into question previous studies examining M1/M2 bias following neurotrauma, we fully acknowledge the role of neuroinflammation in the propagation of neuropathophysiology following TBI. However, we believe that trying to fit highly complex trauma-induced inflammatory molecular mechanisms within the binary constraints of M1/M2 nomenclature are inherently restrictive. To this end, the concurrent differential expression of inflammatory profiles in our current study demonstrates that both semantically and biologically the dogma of ‘polarization’ is not pertinent to TBI. While it is not feasible to perform large profiling experiments for each study, drawing systems-wide inflammatory categorization (e.g. M1, M2a, M2b, M2c, M2d) based upon a few cherry-picked inflammatory markers is not expedient either. This dated dogma should be eschewed in favor of defining the specific regulatory or mechanistic roles of these markers in the context of trauma-induced neuroinflammatory sequelae.
